# An evaluation of the effectiveness of induction programmes on foundation doctor preparedness: a rapid review of the literature

**DOI:** 10.1007/s11845-021-02683-3

**Published:** 2021-06-30

**Authors:** Monika Patel, Jasmine Patel

**Affiliations:** 1 University of Exeter Medical School, Exeter, UK; 2 Ashford and St. Peter’s Hospitals NHS Foundation Trust, Surrey, UK

**Keywords:** Foundation doctor, Induction programme, Medical students, Preparedness, Preparing for practice, Transition

## Abstract

There is an increase in mortality when medical graduates replace the previous cohort of foundation doctors. As of 2012, it is now mandatory for new doctors in the UK to participate in induction training in order to ease this transition and reduce the negative impact on patient outcomes. However, there is no guidance on how best to deliver these induction programmes. This review aims to evaluate the effectiveness of several induction programmes to provide insight on this. Medline and Scopus were searched for relevant literature using keywords. Duplicates were removed and inclusion criteria were created to screen the remaining literature. Five studies were included in this review and they were all quality appraised using the Medical Education Research Study Quality Instrument. Different hospital trusts utilised varying induction programmes. The most common method of assessing their effectiveness involved exploring preparedness in junior doctors post-induction through surveys. Patient outcome, anxiety levels and knowledge were also measured. Induction programmes play a vital role in preparing new foundation doctors for practice and thus improving patient outcomes. Although there may be trust-specific variation, some elements of the programme should be standardised to ensure basic requirements are met universally. New doctors should be assessed on aspects of the programme after completion to increase confidence and knowledge. Organisational considerations such as costs and staff availability need to be taken into account. The quality of future research papers could be improved through inclusion of baseline data, control groups, multi-centred studies and outcomes higher on Kirkpatrick’s hierarchy.

## Introduction

The national changeover day in August, when the previous cohort of foundation year one doctors (F1s) are replaced by medical graduates, has been coined ‘Black Wednesday’ due to increased mortality rates [1]; it has been reported that there is a 4.3–12% increase in mortality in that month alone [2]. This negative impact on patient outcomes is thought to be due to the new cohort being unfamiliar with the hospital environment and with the tasks they are expected to carry out. New doctors often lack confidence and feel unprepared for many aspects of their new role [1]. In light of this, in 2012, the General Medical Council made it mandatory for all new doctors to undergo induction training as the Academy of Medical Royal Colleges recommended a high-quality induction for a safe changeover [3, 4]. However, there is still no national consensus on the content and delivery of the programme, and these differ largely between trusts [5]. The aim of this review is to evaluate different induction programmes (IPs) and assess their impact on junior doctors’ preparedness. Based on the findings, recommendations will be proposed on how to improve and potentially standardise IPs. This will help to inform future programme design and enable doctors to feel better prepared, thus improving patient outcomes.

## Methods

A rapid review was carried out and the Preferred Reporting Items for Systematic Reviews and Meta-Analyses (PRISMA) guidelines [6] were used to increase methodological rigour.

### Search strategy

Relevant literature was searched for on the Medline and Scopus databases on 15th November 2020 by MP. The search strategy and terms of the Medline search using the OvidSp platform are detailed in Appendix Table 2. The same search was conducted on the Scopus database, without ‘Medical Subject Headings’ terms as this is not a feature of Scopus. Forward and backward citation chasing of identified articles was also completed using Scopus (*n* = 9). Duplicates were then removed. This was carried out independently by MP.

### Inclusion criteria

The inclusion criteria below were used to filter the titles and abstracts of the remaining articles (*n* = 900):

- Articles in the English language
- Articles published in the last 10 years
- Studies conducted in the UK
- Primary research papers
- Studies conducted on new F1s
- IPs aimed at preparing new F1s
- One or more quantitative outcomes being measured

**Fig. 1:**
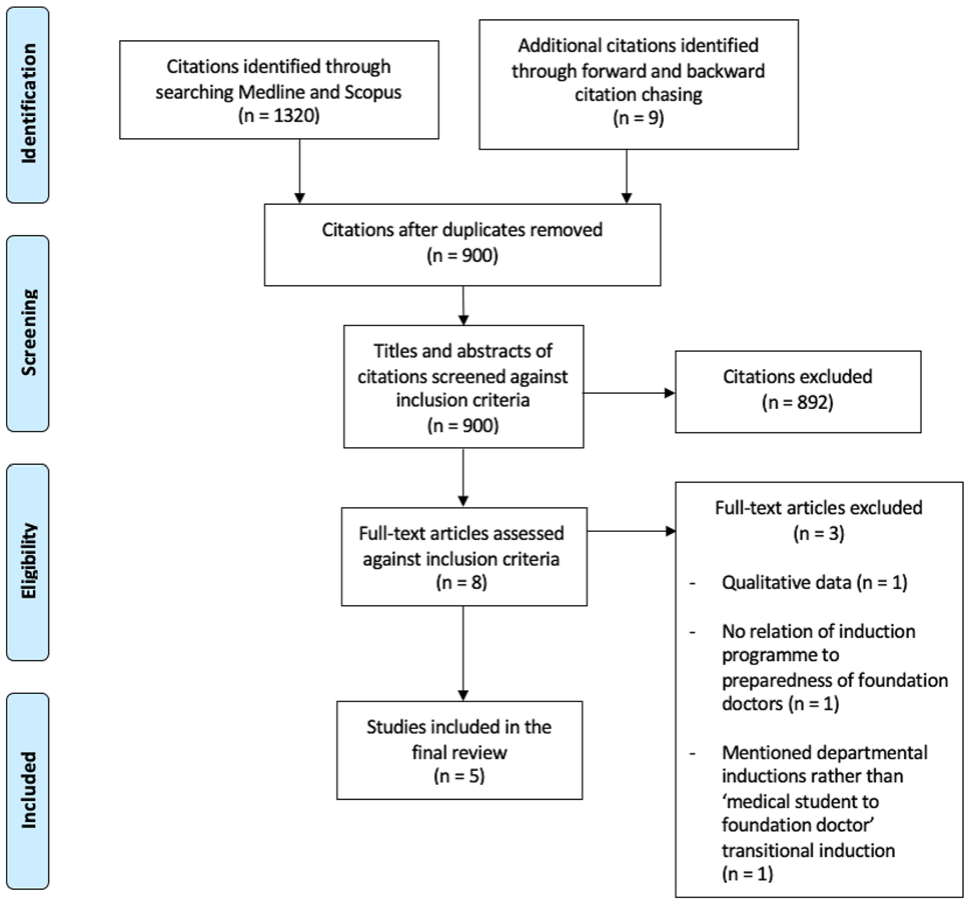
PRISMA flowchart of search process

The remaining articles (*n* = 8) were read in full and screened against the same criteria, leaving five articles to be included in the review. Figure 1 provides an overview of the search process in the form of a PRISMA flowchart [6].

### Data extraction

To ensure only pertinent information was obtained from the five studies, a data extraction form was created by MP and JP, using some components of the Medical Education Research Study Quality Instrument (MERSQI) [7]. It comprises 21 sections, some of which include the number of hospitals involved, duration of IPs and study limitations. The full data extraction form is shown in Appendix Table 3.

To ensure a high level of rigour, the data extraction forms were completed independently by both authors and then compared. There were disagreements in two cases, but these were discussed and eventually resolved.

### Synthesis and quality assessment

Due to the lack of homogenous literature, a narrative synthesis was performed.

The MERSQI was used to appraise the quality of each study [7]. Although the maximum number of points available is 18, the non-applicability of various items in some studies meant they were marked against a lower score. To ensure standardisation, they were converted to a score out of 18, as demonstrated by Merchant and Nyamapfene [8].

There is no consensus on which MERSQI score is deemed “high-quality” but several studies have used 14 as a cut-off [9, 10]; therefore, studies with a MERSQI of below 14 in this review were not deemed high-quality.

As the MERSQI scores were incorporated within the data extraction form, this was also done in duplicate by MP and JP and compared. Only one case caused a disagreement, but this was resolved through discussion.

## Results

### Overview of the studies

Table 1 provides a summary of the five studies included in this review.

**Table 1:** Study overview

	**Author(s) and year published**	**Country of study**	**Induction programme**	**Number of participants**	**Response rate**	**Study design** **A = single group post-test** **B = single group pre-test and post-test** **C = non-randomised 2 groups** **D = randomised control groups**	**Data collection method**	**Key findings of the study**	**Outcome measured** **A = satisfaction** **B = knowledge** **C = behaviours** **D = patient/healthcare outcome**	**MER SQI score (/18)**
1	Sukcharoen et al., 2014	UK	A 10-hour day long near-peer induction	26	96%	B	Questionnaires pre-test and post-test	• Confidence levels increased in prescribing, recognising the critically ill patient and requesting investigations • The most significant increase in confidence was “knowledge of the environment”	A	9
2	Blencowe et al., 2015	UK	Five-day targeted structured induction training before their start date	In 2008: 29 In 2009: 39	In 2008: 74% In 2009: 100% Average: 87%	2 × A	Questionnaire post-test (after 4 months) and self-reported incidents post-test	• In 2008: 83% of F1s felt prepared for their first month. 5 of the 96 self-reported incidents resulted in permanent patient harm • In 2009: 97% felt prepared on their first day. 1 of the 52 self-reported cases resulted in permanent patient harm	A + D	13.2
3	Van Hamel et al., 2015	UK	Assessment of different induction programmes in 22 different UK foundation schools	1829	34%	A	Leeds Self-Assessment of Anxiety General Scale post-test and a survey with a 5-point Likert scale post-test	• Anxiety levels decreased with every day of induction • Confidence and preparedness varied in terms of medical school graduated and foundation school	A	10.8
4	Gaskell et al., 2016	UK	E-induction and e-mandatory training to be completed before their start date (roughly 6.5 hours)	370	100%	A	Multiple choice, post-test summative assessment, and a post-test feedback form	• Foundation doctors passed 90.3% of the mandatory training component assessments • Feedback from F1s was positive	A + B	12.6
5	Thomas et al., 2019	UK	4-page induction booklet	7	71%	B	Survey with a 7-point Likert scale pre-test and post-test	•There was an increase in confidence post-induction • 100% of F1s found the induction booklet useful	A	8.4

All of the studies took place in the UK and were published between 2014 and 2019. Participant numbers ranged from 7 to 1829 with a median of 34. Single group post-test (*n* = 3) [5, 11, 12] was the most common study design, followed by single group pre-test and post-test (*n* = 2) [1, 13]. The most prevalent data collection method was a questionnaire (*n* = 4) [1, 5, 11, 13] measuring confidence or preparedness of F1s starting at a trust, followed by summative assessments testing knowledge (*n* = 1) [12], self-reported incidents (*n* = 1) [5] and anxiety assessments (*n* = 1) [11].

### The effectiveness of the induction programmes

Different foundation schools implemented varying IPs; some consisted of e-induction and e-mandatory training [12], and others used an induction booklet [13]. There were also face-to-face inductions which ranged from five hours to 10 days [1, 5].

The most common method of measuring the effectiveness of IPs involved exploring preparedness in F1s through surveys post-induction (*n* = 4) [1, 5, 11, 13]. Blencowe et al. [5] found that in 2008, 83% of F1s that attended the IPs felt prepared in their first month, compared to 10% of those not attending. In 2009, after induction was made compulsory, this increased to 97%. Sukcharoen et al. [1] measured self-perceived confidence pre- and post-induction; the most significant increase in confidence was in knowledge of the environment, a topic that was heavily focused on in the IP.

Thomas et al. [13] saw an increase in confidence levels amongst F1s through a change in their Likert scale scores for various topics from “difficult” or “somewhat difficult” to “easy” or “very easy” post-induction. However, Van Hamel and Jenner [11] concluded that confidence and preparedness varied depending on medical school of graduation and the foundation school. The same study also showed that F1s’ anxiety levels reduced with each day of induction via the Leeds Self-Assessment of Anxiety General Scale. Gaskell et al. [12] tested the effectiveness of e-learning through a summative assessment post-induction; on average, F1s passed 90.3% of the mandatory training component assessments.

As well as testing preparedness, Blencowe et al. [5] also measured patient outcomes through F1s self-reporting incidents. In 2008, there were 96 self-reported errors, out of which five resulted in permanent patient harm. The risk of a critical incident was higher amongst the F1s that did not attend the IP. Comparatively, in 2009, there were 52 self-reported mistakes, out of which only one resulted in permanent patient harm. That is a 45% decrease in self-reported incidents from 2008 to 2009 post-introduction of mandatory induction.

There were some similarities in the results; many studies showed F1s had increased confidence in requesting the correct investigations post-induction (*n* = 3) [1, 11, 13]. Others demonstrated high confidence at baseline in prescribing intravenous fluids which increased further post-induction (*n* = 2) [1, 13]. Additionally, F1s in 22 different foundation schools in the UK found recognising critically ill patients third most useful out of eight induction topics [11]. This is reflected in Sukcharoen et al.’s study [1], where there was a 9% rise in confidence in recognising critically ill patients post-induction. However, not all results were agreed upon; Sukcharoen et al.’s study [1] showed that familiarity with the e-portfolio increased post-induction. Conversely, there were discrepancies in the familiarity of the e-portfolio depending on the medical school of graduation and the foundation school according to Van Hamel and Jenner [11]. Furthermore, Van Hamel and Jenner [11] found that although there were differences between foundation schools, overall, F1s lacked confidence in prescribing insulin post-induction, whereas Sukcharoen et al. [1] found that confidence levels increased the most (45%) in prescribing insulin.

### Study quality

The mean MERSQI score was 10.8 (range = 8.4 to 13.2). As previously stated, there is no specific cut-off for a “good” MERSQI score, however, several papers have used 14 [9, 10]. Thus, none of the studies were classed as “high-quality”. All of the studies were conducted in a single trust (*n* = 4) except for one that included 22 different foundation schools [11]. The mean response rate of participants was relatively high - 78% (with the range being between 34% and 100%). The outcomes were measured using Kirkpatrick’s hierarchy [14]; all but one study measured the confidence or preparedness of F1s [1, 5, 11, 13], one study measured improvements in knowledge through a summative assessment post-induction [12] and one measured patient outcomes through self-reported incidents [5].

## Discussion

This review aims to evaluate different IPs and proposes recommendations to guide future programme design. This will ultimately increase F1 competence and preparedness, and subsequently improve patient outcomes.

Although each study implemented a different IP, they all still achieved at least one successful outcome. Evidence shows that attending IPs increases F1s knowledge [12], preparedness to practice [1, 5, 11, 13] and reduces the number of self-reported incidents [5]. This illustrates the importance of IPs and considering standardisation so that these positive outcomes can be achieved universally.

Many studies found that allowing F1s to take part in the induction in their own time was preferred as it allowed increased flexibility. Thomas et al. [13] achieved this through an induction booklet, which 100% of F1s found helpful, and Gaskell et al. [12] achieved this through an e-induction which received positive feedback. This adaptability could be introduced into the national IP, thus allowing doctors to work through content in their own time and at their individual paces.

After evaluating the studies, the importance of considering the duration of IPs is evident. Those that participated in Sukcharoen et al.’s study [1] found that the 10-hour day was too intense and that the programme should be spread out over at least two days. There were no comments on the duration of Blencowe et al.’s five-day IP [5], but a longer programme would involve increased costs, organisation, staff availability and more time away from patients. Thus, further research needs to be conducted to find a balance between a longer IP (to optimise knowledge and skills gained by F1s and subsequently increase preparedness) and the costs incurred. However, it could be argued that the benefits of a longer IP in improving patient outcomes and potentially reducing mortality rates outweigh the limitations of any costs or organisational issues.

Although measuring patient outcomes through F1s self-reporting errors sounds promising, this relies upon doctors recalling and honestly reporting these. In Blencowe et al.’s study, critical incidents were confirmed by the hospital reporting system, however, non-critical errors may have been overlooked. Furthermore, the study was only carried out during the first four months of the foundation year; this reduction in errors may not have been maintained throughout the year. With under-reported incidents and no long-term data, it is difficult to ascertain to what extent IPs actually help patient outcomes by reducing F1-led incidents [5].

Confidence levels in recognising critically ill patients were high amongst F1s post-induction [1] and most found teaching around this useful [11], however, no IPs have tested their ability to manage such patients [1]. In fact, research shows that new F1s may actually have scarce understanding of this [15]. This questions whether the high confidence levels are due to their recently passed final exams, rather than the actual ability to recognise and potentially manage a critically ill patient.

Additionally, although studies show that post-induction, F1s found requesting the correct investigation “easy” [13] or felt more confident in doing so [1], there was no formal assessment to confirm this. Although testing every skill is not feasible, some form of testing should be considered as confidence is subjective and does not reflect on actual knowledge or skills. Despite this, only one out of the five studies held a post-test assessment [12]. An online simulation could perhaps be carried out to test skills deemed “most important”.

There were clear discrepancies between studies on the confidence F1s had in prescribing insulin [1, 11], further reinforcing the need to consider standardisation of IPs in the UK. Teaching should be effective enough for all F1s to feel confident in prescribing drugs such as insulin; this may be improved by perhaps having the topics taught by clinicians experienced in teaching.

There were also conflicting studies [1, 11] around how familiar F1s were with their e-portfolio and the requirement to maintain it. This perhaps shows a lack of e-portfolio training in some IPs and is an area that needs to be incorporated in every IP. This is especially important as e-portfolios are not something that F1s have previously learnt about as medical students. It is also a possibility that the low response rate in Van Hamel and Jenner’s [11] study affected its results.

Furthermore, participation in some IPs included in this review was optional (*n* = 4) [1, 5, 11, 13] suggesting that the participants that provided feedback may have been those with strong viewpoints. Some studies [11] also had a low response rate hence do not represent the entire year group.

The benefit of evaluating different IPs is that it enables trusts to be better informed on how to deliver induction in a way that works best in their setting. Although it is understood that different trusts have different needs, it is clear that some form of standardisation is still needed to tackle important areas such as insulin prescribing and e-portfolio training. A basic induction is proposed to ensure that all new doctors meet the minimal standards, however, trust-specific requirements also need to be fulfilled. Further research needs to be conducted to determine which areas should be standardised.

According to the MERSQI scores, none of the studies were classed “high-quality”, mostly due to methodological limitations. Three out of five studies [5, 11, 12] did not have robust results as they did not include any baseline data to compare to; this made it difficult to measure the effectiveness of each intervention. None of the studies had control groups which reduced validity as there could have been other factors influencing the findings. It is therefore unknown whether any changes detected occurred due to the intervention or due to other factors. Thus, to optimise results in future studies, control groups would need to be implemented, including both pre-tests and post-tests, in order to ascertain the effectiveness of each intervention.

Moreover, no studies had outcomes measuring F1 behaviour and only one measured patient outcomes [5], although only in the short-term. Both of these outcomes could have been measured long-term through a longitudinal study design; the participants could have been followed up after a year to observe any improvement in outcomes. This is, however, difficult to implement due to high attrition rates and the burden on participants [16, 17].

Finally, four out of five studies [1, 5, 12, 13] were single-centred meaning they may not be representative of the whole F1 population, thus reducing transferability of the results. In the future, to improve this, multi-centred studies would need to be implemented.

## Strengths and limitations

The transparency of the search strategy and the use of specific inclusion criteria are strengths of this review as they ensured that only relevant studies were included. The key findings of each study were also synthesised in order to propose recommendations on how to improve IPs to better prepare F1s, thereby improving patient outcomes. Additionally, the use of the MERSQI to appraise the quality of data was beneficial as it allowed studies to be compared due to its standardised format.

To keep this review current, only articles from the last 10 years were included. This, however, limited the study as key findings of older studies may have been missed. This review could also have been subjected to publication bias due to the fact that grey literature was not searched; this may have led to the omission of vital data from unpublished studies. Moreover, inclusion of studies only in the English language could have potentially excluded useful interventions published in other languages. Furthermore, only two databases (Medline and Scopus) were searched which reduces the scope of the study.

Finally, the lack of baseline data also limited the study as it did not allow a standardised framework to be used to measure the effectiveness of the IPs, such as the one utilised by Gill et al. [18]. Gill et al.’s framework measures the percentage change of the outcome from the baseline to post-test, in order to establish the effectiveness of the intervention. As only two studies [1, 13] had pre-test data, the effectiveness of each IP could not be measured and compared.

## Conclusion

IPs are a necessity to prepare F1s for practice and improve patient outcomes, and some form of standardisation should be incorporated to ensure basic F1 requirements are met universally. Due to the paucity of literature surrounding IPs, it is difficult to suggest recommendations on the duration and content of all IPs nationally. However, studies have suggested that incorporating an online component is beneficial as it allows F1s to complete aspects of it in their own time. If possible, F1s should be tested on elements of the programme after completion to ensure understanding and knowledge. Costs, staff availability and time away from patients need to be considered when implementing this. The quality of future research papers could be improved by including baseline data, control groups, multi-centred studies and higher outcomes on Kirkpatrick’s hierarchy. Due to the remarkable increase in mortality rates in the changeover month alone, future research on IPs should address patient outcomes with a particular focus on mortality rates. The impact of medical schools on F1 preparedness to practice could also be explored, to further improve patient outcomes.

## Appendix

**Table 2:** Medline search strategy

	Terms searched
1	foundation doctor*.ti,ab
2	junior doctor*.ti,ab
3	new doctor*.ti,ab
4	trainee doctor*.ti,ab
5	foundation year*.ti,ab
6	fy1.ti,ab
7	f1.ti,ab
8	(foundation adj2 doctor*).ti,ab
9	(junior adj2 doctor*).ti,ab
10	(new adj2 doctor*).ti,ab
11	medical student*.ti,ab
12	medical school*.ti,ab
13	induction*.ti,ab
14	induction program*.ti,ab
15	(induction adj2 program*).ti,ab
16	induction course*.ti,ab
17	(induction adj2 course*).ti,ab
18	induction train*.ti,ab
19	(induction adj2 train*).ti,ab
20	(induction adj3 train*).ti,ab
21	prepar*.ti,ab
22	improv*.ti,ab
23	skill*.ti,ab
24	knowledge.ti,ab
25	assess*.ti,ab
26	confiden*.ti,ab
27	exp Clinical Competence/
28	1 or 2 or 3 or 4 or 5 or 6 or 7 or 8 or 9 or 10 or 11 or 12
29	13 or 14 or 15 or 16 or 17 or 18 or 19 or 20
30	21 or 22 or 23 or 24 or 25 or 26 or 27
31	28 and 29 and 30

**Table 3:** Data extraction form

1	Inclusion criteria still met? If not, why?
2	Title of paper
3	Author(s)
4	Journal and year published
5	Aim of the study
6	Study design (A = single group cross-sectional/single group post-test, B = single group pre-test and post-test, C = non-randomised 2 group, D = randomised control trials, other = please state)
7	Country study carried out in
8	Number of hospital trusts involved
9	Number of participants
10	Response rate (%)
11	Duration of induction programme
12	Description of induction programme
13	Topics included in the induction programme
14	Outcome measured (A = satisfaction/attitudes/perceptions/opinions, B = knowledge/skills, C = behaviours, D = patient/healthcare outcomes)
15	Main findings
16	Main conclusions
17	Avenues for further research
18	Study limitations
19	Related references that could be useful
20	Medical Education Research Study Quality Instrument (MERSQI) score (/18)
21	Other comments
